# Progress in State Medicine

**Published:** 1903-06-20

**Authors:** 


### Progress in State Medicine.

### HOUSING OF THE WORKING CLASSES.

Fyfe[1] defines a "slum" to be "a congeries of dwelling-houses situated in such close contiguity to each other that free access of air and good light to all or some of them is prevented, where nuisance must be constantly caused by the close proximity of ashpits and conveniences, and where the internal arrangements do not admit of adequate or proper ventilation." The houses make the slums, the tenants constitute the danger of them. The City Improvement Trustees of Glasgow, and the Glasgow Workmen's Dwellings Company, define as "the poor" those who earn less than 26s. per week—an unsatisfactory classification, because a single man is in a better position than a married man with the same wages, and a married man with no family is considerably better off than one who has several children to support. To define "poor people" it is necessary not to look at wages only, but what those wages have to do. Fyfe would define the poor as "all those persons whose average weekly wage does not enable them to maintain themselves and their dependents up to a reasonable standard of physical efficiency after expending one-tenth of their wages in house rent, and laying past 10 per cent. of their earnings for contingencies." In Glasgow, and other cities, the poor, as a rule, dwell in one and two-apartment houses in the congested areas, owned by those who let them their houses for as much profit as they can get, and generally at rents, in Glasgow, of 2s. per week for one-apartment houses, and 2s. 9d. per week for two-apartment houses, taking averages. These houses, known in Glasgow as "ticketed" houses are not healthy. Thousands of them have no value as dwellings, their present value being entirely due to the want of wholesome houses at a price within the means of those defined as "poor." In support of his belief that such houses can be provided at a profit he quotes the authority of Mann, Secretary to the Glasgow Workmen's Dwelling Company, who says : "I therefore state it as my deliberate opinion that plain and sanitary houses built on ground costing 20s. per yard, managed efficiently, and let at rents similar to those charged for the average "ticketed" house, will yield the builder a return of 3 per cent. to 3¼ per cent. ; while if a 2⅜ per cent. suffices 30s. per yard, or even more, may be paid for the ground. Fyfe considers the private builder quite unfit, from a commercial point of view, to house even selected poor. Philanthropy would do more but for the known reluctance of the poor to leave the centre of bustle and excitement of town life for the adjoining suburbs where land is cheaper. Further, city residence for the poor is entirely in favour of their financial interests. This entails that either travelling facilities must be greatly increased and cheapened to the suburbs, or that municipalities must provide such dwellings in the towns, and be content with 3 per cent., or 3¼ per cent. on the money expended. The most difficult problem is how to provide for the incorrigible, vagrant, vicious, non-rent-paying poor, who constitute about 25 per cent. of the total poor. The only course is to seek powers from Government to place all such in reformatories or working colonies, where work must be done, and where they must remain until a commission is satisfied there is a reasonable prospect that reformation has set in. The cost of these reformatories would be partially maintained by Government and partially by the communities sending the people in. The dens which they presently inhabit should be, if found insanitary, closed against human habitation, or made sanitary and wholesome for corrigible, decent citizens. Bond [2] considers that the whole difficulty of the housing question is the difficulty experienced by the working classes in finding suitable accommodation for their requirements within their means. In London, in other respects, things are a great deal cheaper than they were 10 or 15 years ago, but house rent is not cheaper, partly on account of increased cost of construction. Efforts to provide suitable accommodation have been hampered by mistaken legislation and faulty administration. The law required alteration so that undesirable tenants could be promptly got rid of, there being a class of people going about who practically, by a knowledge of the law, were enabled to live almost rent free for the greater part of the year. Samuel [3] emphasises the importance of providing cheap and rapid travelling facilities with the suburbs. The rents are affected by the high price of land, the value of which in cities is always increasing. The period of loans for land might well be made 100 years, and that for the repayment of the building loan 80 years. This would make a difference in the rent of dwellings of as much as 6d. per room per week. Not only are houses wanted in cities, but also in villages, where the accommodation for the labourers is often of the poorest description. Fyfe,[4] in his evidence before the Glasgow Housing Commission, stated that there were 13,914 one-apartment, and 6,005 two-apartment "ticketed" houses in Glasgow. They housed about 74,000 persons, or nearly a tenth of the total city population. In the 30 years from 1871 to 1901 the proportion of one-roomed houses had fallen from 30 per cent. to 15 per cent. These houses occurred for the most part in congested areas, dotted all over the city. The average number of persons to the acre for the whole city was 60, but in these congested areas there were from 508 to 876 persons per acre, and in some of the smaller blocks the rate went up to 1,000 persons to the acre. There were 3,394 houses which required to be dealt with under the Building Regulations Act, housing 14,000 people, for whom it would be necessary to provide sanitary dwellings at as nearly as possible equivalent rents to those which they at present paid. He stated it as his opinion that it would not be expedient to carry out any appreciable measure of closing until provision was made for the population of the displaced. Dealing with the character of the tenants of these 3,894 houses, 2,494 were occupied by respectable tenants, 935 by more or less drunken tenants, and 467 by the vicious and criminal class. Ventilation was deficient in 4·9 per cent. of the total, and 6·1 per cent. were deficient as to light. The greater number of the people to be displaced were respectable but very poor, and at the outside they could not pay more than £6 a year rent. Private enterprise being unable to build houses of the necessary description on commercial lines, it fell to the Corporation, supplemented by semi-philanthropic agencies, to take the matter up. To build 3,894 houses of one and two apartments would cost £455,598, while suitable sites could be obtained at a less average cost than 20s. per square yard, and for this purpose £129,600 would in addition have to be set aside. Munro, before the same commission, stated that if people were housed in so grossly insanitary a way as to be a menace to the public health, and no other provision could be made for them then the Local Authority was bound on principle to find the accommodation. There would be no rush to the Corporation property by these people, as they were too fond of their independence. But having provided houses the Corporation should harry them from place to place, until they either got them to occupy decent houses, which they would keep decently, or sweep them into the Corporation houses. He suggested that in addition a man should be liable to be punished, and that not merely by fine, for failing to provide a wholesome house for his family when able to do so. Attention [5] is called to the fact that a few years ago efforts were chiefly directed to clearing slum areas ; now the greater importance of first providing new houses and cheap means of communication is fully realised. Taylor advocated, in the recent discussion in the House of Commons, that the Local Government Board, when a slum was cleared, should insist not only on substitutory accommodation, but should also require that the accommodation should be allocated to those who were dispossessed. It is pointed out also that the experience of London and other large towns in clearing slums is not likely to favour further extensive experiments in this direction. During the last 27 years 34 slum areas have been cleared in London at a cost of £3,000,000, of which £2,000,000 is stated to be an absolute loss to the ratepayers of the metropolis. Three separate Bills [6] are at present before the House of Commons in connection with the subject of the housing of the working classes. (1) The Housing of the Working Classes (London) Bill, which has for its object the regulating of the conditions under which displacement of persons of the labouring class may be made in London under statutory powers, and the requiring of sufficient new housing accommodation. (2) Housing of the Working Classes (Repayment of Loans) Bill. This is a Bill to extend the period for repayment of loans relating to the housing of the working classes, and for other purposes connected therewith. In determining, under the provisions of this Act the period for the repayment of any moneys borrowed, the sanctioning authority may, in its discretion, prescribe a period not exceeding three years from the date of the borrowing, during which the first payment into the sinking fund or the payment of the first instalment may be deferred. (3) Housing of the Working Classes. This is a Bill to amend the law relating to the housing of the working classes, and to establish fair-rent courts. One of its objects is to assist local authorities to deal with the housing question by obtaining loans from Government at 2 per cent. interest. The maximum period for repayment is at present 60 years, which it is proposed to extend to 100 years. It also provides that any land that may be acquired under the provision of the Housing Acts may be retained and held by the local authority for a period of 20 years from the date of purchase, without being built upon. The site [7] of the new dwellings, in course of erection by the Council of the City of Westminster, contains nearly an acre and a half, and covers ground formerly occupied by factories and dilapidated house property. The buildings consist of three parallel blocks six storeys in height, and can house 344 families, or 1,600 persons. The rents have been fixed at a point lower than those ruling in the neighbourhood. The weekly rent for a one-room tenement will be from 3s. to 4s., two-room tenements 5s. 6d. to 6s. 6d., three rooms 8s. to 9s., and four rooms 11s. to 12s. The sanitary arrangements are as perfect as they can be made, and laundries, drying-rooms, bath-rooms, and urn-rooms—the latter for the supply of hot water at meal times—are provided. As an experiment, coal will be sold to the tenants at practically cost price. The rents have been adjusted to a scale that will, after permitting for a sinking fund to repay the total outlay, give a net return of $3\frac{1}{2}$ per cent. per annum.

[1] Sanit. Rec., Oct. 9, 1902. [2] Sanit. Rec., Jan. 1, 1903. [3] Ibid. [4] Ibid. [5] Brit. Med. Jour., April 11, 1903. [6] Sanit. Rec., April 23, 1903. [7] Pub. Health Engineer, May 2, 1903.

